# Mechanical Reclamation of Spent Moulding Sand on Chromite Sand Matrix; Removal of Alkali-Phenolic Binder

**DOI:** 10.3390/ma16072919

**Published:** 2023-04-06

**Authors:** Mariusz Łucarz, Aldona Garbacz-Klempka, Dariusz Drożyński, Mateusz Skrzyński, Krzysztof Kostrzewa

**Affiliations:** 1 Faculty of Foundry Engineering, AGH University of Science and Technology, Reymonta 23 St., 30-059 Krakow, Poland; 2 Huta Małapanew Sp. z o.o., 1 Kolejowa, 46-040 Ozimek, Poland

**Keywords:** foundry engineering, mechanical reclaim, chromite sand, moulding sand properties, scanning electron microscopy, thermal analysis

## Abstract

The foundry industry generates large amounts of waste when casting metal into sand moulds. An important issue is the activities that are related to the re-recovery of the grain matrix (the main component of the moulding sand) for realising subsequent technological cycles. This process is particularly important in the case of the expensive chromite matrix that is necessary for use in manganese steel casting. The effects of the reclamation treatments of spent alkali-phenolic binder sand were evaluated by scanning electron microscopy with EDS, analysing the chemical composition in micro areas and proving the loss of binder on the surfaces of the matrix grains. Tests were also performed using the main criteria for evaluating a reclaimed organic binder: sieve analysis and ignition loss. A thermogravimetric analysis study was performed to assess the change in the chromite character of the grain matrix under the influence of temperature. The effects of the reclamation measures were verified by making moulding compounds on a matrix of reclaimed sand and a mixture of reclaimed and fresh sand. The tests and analyses that were carried out indicated the direction of an effective method for reclaiming used alkali-phenolic binder masses and the extent of the proportion of the regenerate in moulding sand in order to maintain the relevant technological parameters of the moulding sand.

## 1. Introduction

The production of steel castings that use the alkali-phenolic Alphaset process of moulding sand preparation has been recognised as the most convenient and environmentally friendly casting process and has become the standard in England [1]. The benefits of the alkali-phenolic process are a significant improvement in casting quality and a reduction in the costs that are associated with casting cleaning. At the same time, the total amount of gaseous substances given off when a mould is flooded with liquid metal is significantly lower than with other organic resins [2]. The tensile strength of a moulding compound that is prepared on a matrix of new sand using alkali-phenolic technology is generally lower than that of other organic resins [3]. However, the tensile strength is sufficient for meeting the requirements of core and mould technology in most applications while improving the surface quality of the casting [4]. The main advantage of the alkali-phenolic process is the two-stage setting—an initial setting at the ambient temperature, and an essential setting from the temperature of the liquid casting melt. The initial strength that is obtained is sufficient for carrying out the technological procedures that are associated with the casting mould. The alkali-phenolic resin (which is not fully bonded) exhibits thermoplastic properties; this compensates for the resulting thermal expansion of the grain matrix during the pouring of the mould with liquid metal, eliminating mould cracking and the possible outflow of liquid metal. At the same time, the high-dimensional accuracy and resistance to penetration of the liquid casting alloy is achieved [5,6].

The authors of [7] compared bonding systems for large-sized castings, stating that taking into account the increasing environmental requirements, activities should be directed to the development of optimal properties of moulding compounds for casting production. This is an important issue for the European foundry industry, which sees a solution for this situation in the use of Alphaset technology [7]. Therefore, alkali-phenolic resin is replacing furan resin in cast steel foundries for ecological and economic reasons [8]. The results of studies on the use of the Alphaset alkali-phenolic resins from different manufacturers and using different grain matrices were presented by the authors in [9]. They concluded that, based on comparative studies, a suitable alkali-phenolic resin could be identified (adopting the criterion of the highest moulding sand strength) that is predisposed as a substitute for furan resin. However, this is a difficult process that requires a complete replacement of the circulating regenerate (acidic reaction) with a matrix sand that will be alkaline in nature.

According to resin manufacturers’ recommendations for the alkali-phenolic process, one to two parts of the highly alkaline phenolic (resol) resin by weight is added to the grain matrix and, in the next step, about an 18 to 25% aliphatic ester-to-resin ratio. For chromite and olivine sand, more resin is added (about 2 parts by weight), and the ester is added at a resin-hardener ratio of 5/1. Depending on the type of ester that is used, the curing time can be adjusted from 5 to 30 min [10,11,12,13]. 

The difference between the use of NaOH and KOH in Alphaset resin formulations is being considered in foundries. According to the opinion that was presented in [8], the author believed that the achievement of the required strength by a given binder was dependent on the formulation and process parameter and not the type of principle that was used. At the same time, the author pointed out that it takes a lot of skill to create the right procedure in a given foundry.

Systematic studies that have used different reclamation methods and different equipment to reclaim used moulding sands from the alkali-phenolic process have indicated that one of the reasons for the poor binder properties of the reclaimed sands, that could be observed in earlier sands from the alkali-phenolic process, was the presence of a thin layer of inorganic compounds on the surface of the grain matrix. When recovering the grain matrix after the alkali-phenolic process by mechanical methods, it should be borne in mind that the alkali metals that are contained in phenolic resins can react with the sand surface during the moulding. Alkali salts can alter the chemical reaction on the sand surface and remain on the grain matrix [3]. The proportion of regenerate after the mechanical process is no more than 70%; however, supplementing the mechanical reclamation with a thermal treatment increases the proportion of the regenerate in the grain matrix of the prepared mass to 95%. The alkaline salts that are formed have a greater effect on the grain matrix after thermal reclamation than in the case of mechanical reclamation; they can react with quartz sand to form a silica glaze. Sintered silicates reduce the yield after the reclamation process. In addition, sodium and potassium salts can accumulate in the reclaimed grain matrix and reduce the tensile strength. To prevent this from happening, special additives (0.6–1.0%) are added to the knocked-out spent matrix just before the thermal reclamation process. The mineral additives that are used minimise the sintering temperature of the compounds. The introduced additive combines with the alkali metals and is removed in the form of fine particles during the pneumatic classification process [8].

The results of studies on alkali-phenolic binder mass on a quartz sand matrix were presented in [9,14]. As far as chromite sand solutions are concerned, it is difficult to find studies that address them. This is for the obvious reason that chromite sand is more expensive, hence quartz sand is more commonly used. However, for steels with higher melting points (especially in the so-called thermal junctions) as well as massive castings in which a lot of heat energy is accumulated (or when the quartz matrix reacts with a particular type of steel; e.g., manganese [15,16]), it is necessary to use a matrix with a higher melting point (quartz sand—1713 °C; chromite sand—1870 °C [16]) and a correspondingly higher sintering temperature (quartz sand—1350 °C; chromite sand—1500 °C) in order to obtain good castings.

According to the PN-91/H-11007 Polish standard [17], the composition of chromite sand should contain 46% Cr_2_O_3_, 1.5% SiO_2_, 26% Fe_2_O_3_, 15% Al_2_O_3_, 0.1% CaO, 10% MgO, and the rest water. The shape of the grains is mostly angular. Chromite sand is black, slightly greyish in colour, has a density of 4500–4800 kg/m^3^. The grains of this material are brittle, which results in an angular shape. Its sintering temperature is within a range of 1350–1500 °C; also, the pH of chromite sand is within a range of 7–10 [16].

The changes that occur on the grain surface of the chromite matrix are presented in [18]. The authors found that, as the temperature increases, the grain matrix-formed layer of iron oxides becomes less and less bonded to the grain, resulting in easier chipping of these areas during the processing of moulding sands. The phenomenon of the brittleness of the surface layer and the exposure of the grain material may favour the reclamation of the waste base of moulding sands that contain sodium silicate. This is a favourable situation for this binder. The issues of changes on the grain surface of the chromite matrix are also described by the authors in [19].

The use of different types of binders that are used to produce cores and moulds was discussed in [20]. The author pointed out that an important feature of their manufacture should be dimensional accuracy—especially those of cores. Research is being conducted with different resins that aim to optimise the composition in terms of dimensional accuracy. In [21], the authors conclude that the Alphaset system had a higher environmental acceptability; emissions from the Alphaset system were two to five times lower than those from furan resin moulding sand (no-bake). This important environmental and economic aspect (the price of chromite sand) determined the decision to research the influence of a mechanical reclamation procedure on a spent sand that was prepared on a chromite matrix with an alkali-phenolic binder. This article presents the changes that occur on the surface of the grains in the relationship between these two materials as a result of mechanical reclamation and the supplementary effect of the temperature that is applied in assessing the quality of the regenerate (ignition losses). The process of reclaiming spent moulding sand was described in [22,23,24,25,26,27,28,29,30,31]. The mechanical reclamation of used grain matrix was addressed in [32,33,34]. Issues that are related to thermal reclamation (the thermal destruction of organic binders on the grain matrix) are presented in [35,36,37,38,39,40,41].

## 2. Materials and Method

The study used an alkali-phenolic resin with the trade name of Permabind 440. One of the same manufacturer’s range of dedicated Permabind P8 hardeners was used to bind the resin. The respective proportions of the ingredients that were used are shown in Table 1.

The main research consisted of comparing the chemical compositions of fresh chromite sand (CS), spent moulding sand (SS) from the Małapanew Ozimek foundry, and the grain matrix that was obtained after 5, 10, and 15 min of reclamation (MR5M, MR10M, and MR15M, respectively). The quality of the fresh matrix and the regenerates that were obtained were the bases for comparing the technological properties of the moulding sand with a matrix of fresh chromite sand with that which was made on a matrix of regenerate after 15 min for different proportions of regenerate in the moulding sand (MR15M25CS75—regenerate MR15M [25% share], sand matrix CS [75% share], MR15M50CS50—regenerate MR15M [50% share] and sand matrix CS [50% share], MR15M75CS25—regenerate MR15M [25% share] and sand matrix CS [75% share], MR15M100—regenerate MR15M [100% share], SS100—spend sand [100% share]). Thermal analysis studies on the individual grain matrices and the ignition loss were carried out to verify the reclamation treatments that were carried out. The aim of the undertaken experiments was to determine what amount of regenerate could be introduced into the mass without losing its basic technological properties. The compositions of the masses that were tested are shown in Table 1. 

Several mixtures of grain matrices were prepared for testing in accordance with the planned cycle on the effective use of sand and spent sand (burnt and crushed) as well as pre-reclaimed mechanically used moulding sand in the production of cast steel. 

**Figure 1:**
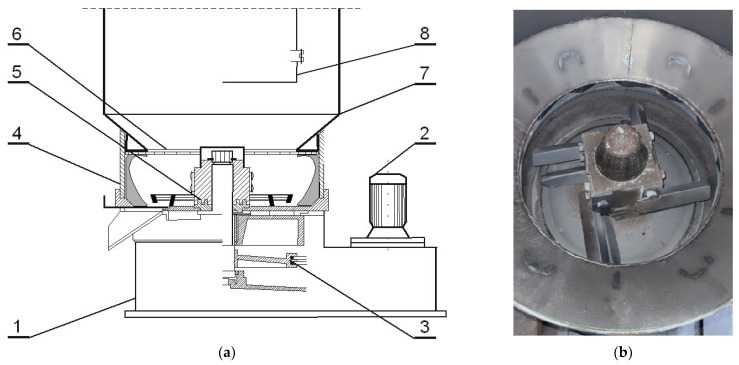
Experimental Rd-6 rotor regenerator: (**a**) schematic diagram [1—base of device; 2—drive motor; 3—belt transmission; 4—regenerator circular ring; 5—rotor with abrasive and crushing elements; 6—air guide; 7—dust-collection chamber casing; 8—inspection door]; (**b**) inside regenerator.

The regenerates (which were designated as MR5M, MR10M, and MR15M) were obtained by the reclamation of the grain matrix (spent sand—SS) in an RD-6 mechanical rotor regenerator (Figure 1) [42]. The machining operations were carried out on a 6 kg portion of the charge for 5, 10, and 15 min, respectively, at a centrifugation speed of 1150 rpm. The rotating rotor of the regenerator induced the movement of the spent sand, which resulted in elementary cleaning operations (abrasion and rubbing).

The first test was a sieve analysis of the individual grain matrices that were obtained as a result of the implemented treatments. The analysis was performed according to the PN-83/H-11077 standard [43] on a set of sieves with mesh clearances of 1.6, 0.8, 0.63, 0.40, 0.32, 0.20, 0.16, 0.10, 0.071, and 0.056 mm (and bottom). The test was performed on two parallel 50 g samples. An LPzE-2e laboratory shaker from Multiserw-Morek was used. The sieving time was 15 min.

Another study was to assess the quality of the grain matrix by observing the grain surface for its shape and the presence of resin residues. The study was realised by scanning electron microscopy (SEM) using a Tescan Mira high-resolution microscope with an FEG electron source. The topography of the sample was studied using solid-state detectors; the beam energy was 20 keV. “Low Vacuum” mode (Chamber Pressure 40 Pa, Injected Gas N_2_) was used in the imaging. The surface was observed in backscattered electron contrast (BSE and BSE COMPO), thus obtaining a material contrast that facilitated the differentiation of the grain components and resin residues (according to each atomic number Z of the elements). Investigations were carried out at various magnifications. The chemical composition was analysed in selected areas by energy dispersive X-ray spectroscopy (EDS) using an Ultim Max EDS detector from Oxford Instruments (Abingdon, UK). The Ultim Max detector belongs to the generation of silicon drift detectors (SDD) that, when combined with Extreme electronics, delivers speed and high sensitivity analysis. Additionally, the AZtec Energy system provides advanced analytical functionality. Energy-dispersive X-ray spectroscopy (EDS) is an analytical technique that can be successfully applied for the elemental analysis or chemical characterisation of a sample in its macro and micro areas. It should be noted that a precise determination of the contents of elements with low atomic numbers is difficult [44,45,46,47]. All of the chemical analyses that were performed on the tested materials were carried out under the same measuring conditions and parameters. The chemical composition was analysed both in selected areas and as a map of the distribution of the elements on the grain surface. Studies were carried out on representative areas at 1000× magnification.

The alkali-phenolic binder residues on the surfaces of the matrix grains were assessed by performing a thermal analysis test. The oven was heated at a rate of 10 °C/min to a temperature of 1000 °C, at which the organic compounds should have been fully degraded and destroyed. The test was carried out using a TA Instruments (New Castle, DE, USA) SDT Q600 thermal analyser (DSC/TGA). The mass of the samples that were subjected to TG thermal analysis was approximately 20 mg for resin and 2 g for grain matrixes. Aluminium oxide crucibles were used for the measurements, which allowed for measurements up to 1500 °C.

An important parameter for evaluating the grain matrix after the mechanical regeneration was the ignition loss. Materials in bulk form were subjected to roasting in an SNOL 8.2/1100 resistance furnace on two samples that were weighed into quartz crucibles. The results that are presented in this paper are the averages of the results that were obtained. The determination was carried out under the following conditions: heating and cooling with a furnace; a heating temperature of 1000 °C; a heating time of 2 h.

The prepared grain matrices were used to carry out a moulding compound tests for different proportions of fresh matrix and regenerate at a constant dosage of resin and hardener (according to Table 1). All of the masses were prepared in a Labor Mischka 00GF/79 Vogel & Schemmann Maschinen GMBH paddle mixer. To a given grain matrix, the hardener (Permabind P8) was added first and mixed for 60 s, followed by the resin (Permabind) and mixed again for 60 s.

The mass that was prepared in this manner was poured into moulds that reproduced the shapes for determining the mass properties that were tested. The moulds for the testing were made on an LUZ-2e machine (Multiserw-Morek, Brzeźnica, Poland) and compacted for 15 s with a vibration amplitude of 2 mm. The prepared shapes were set aside to cure under ambient conditions. Eight-sided shapes were prepared for testing tensile strength R_m_^u^, longitudinal shapes for testing bending strength R_g_^u^, and cylindrical shapes for testing permeability P^u^ and abrasion S. 

The tensile strength R_m_^u^ and bending strength R_g_^u^ of the cured fittings were determined according to the PN-83/H-11073 standard [48]. Measurements were carried out on an LRu-2e universal strength-determination apparatus made by Multiserw-Morek after three curing times (setting off of the compacted fittings): 1, 3, and 24 h. After each curing time, the strength was tested on the three fittings; the obtained results are presented in the graphs as the arithmetic means of the measurements that were taken.

The permeability of cylindrical P^u^ samples in a hardened state was measured based on the PN-80/H-11072 standard [49]. The determinations were carried out using a hasty method on an LPiR-3e apparatus from Multiserw-Morek. The hardened cylindrical sample was mounted in a special sleeve with an inflatable rubber gasket inside (pressed against the side of the moulded part) by using compressed air from a hand pump. The permeability for each mass was determined on the three fittings after being cured for 24 h; the results that are shown in the graph are the arithmetic averages of these measurements.

The abrasiveness of the tested sands was measured on a device that was manufactured by Huta Stalowa Wola in accordance with BN-77/4024-02 [50]. The measurement consisted of mounting a weighed cylindrical sample (hardened for 24 h) that was made of the tested sand in an apparatus holder. The piece was then rotated at a speed of 1 rps with an electric motor. During the experiment, a 307 mm shot was dropped onto the rotating specimen, causing abrasion. The steel shot had a diameter of 1 mm and weighed 1750 g (weighed to an accuracy of 1.0 g). The measurement was carried out on three samples; the results that are presented in the graph are the arithmetic means.

**Figure 2:**
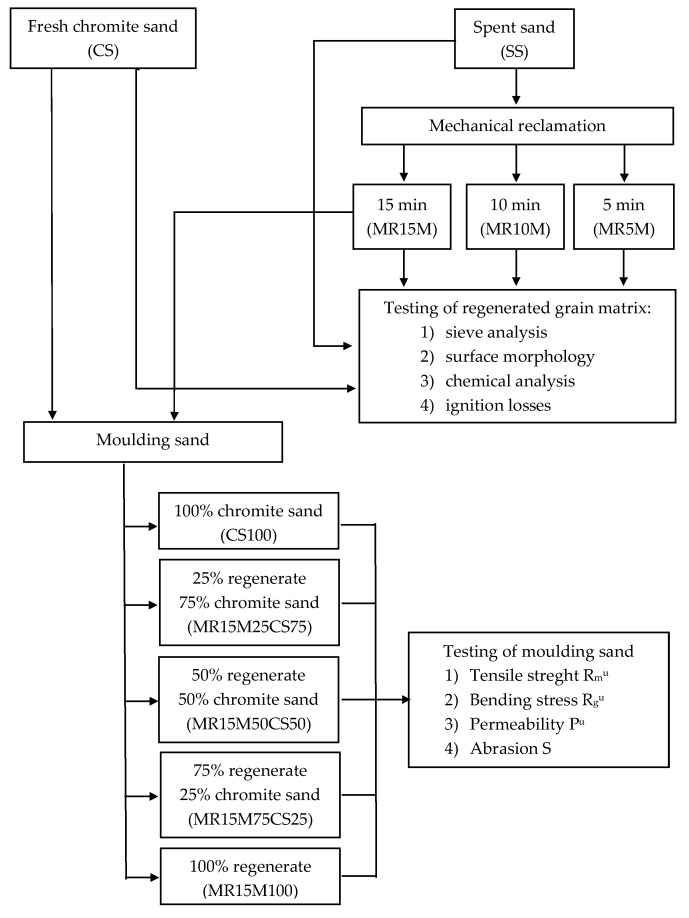
Flow chart of methodology.

Figure 2 shows a flow chart of the methodology.

The tests were conducted under the following ambient conditions: temperature T_ot_ = 14.8–15.4 °C; relative humidity W_w_ = 28–33%.

As part of the analysis of the influence of the mechanical reclamation process on the quality of the matrix, a gas-forming test was also performed. This measurement consisted of heating a tubular furnace (a quartz tube) to a temperature of 1000 °C; a ceramic boat with a 5 g sample of the tested material (weighed to an accuracy of ±0.001 g) was then introduced (outside the heating zone). The results of the measurements that were taken were converted to one gram of sample mass. Three measurements were taken for each sample. After the analysed material sample was introduced, the tube was sealed on one side and connected to a peristaltic pump on the other in order to create negative pressure in the reactor and pump out the resulting gases. The sample was then introduced into the heating zone, where it was heated very quickly to the measurement temperature, and the amount of gas that was formed was recorded. The gas-forming properties of the material samples were measured on a bench that was equipped with a PRC 30M/1300 tubular furnace from CZYLOK, a BT100-2J peristaltic pump from LongerPump, and a control and recording unit [9,10].

## 3. Results

**Figure 3:**
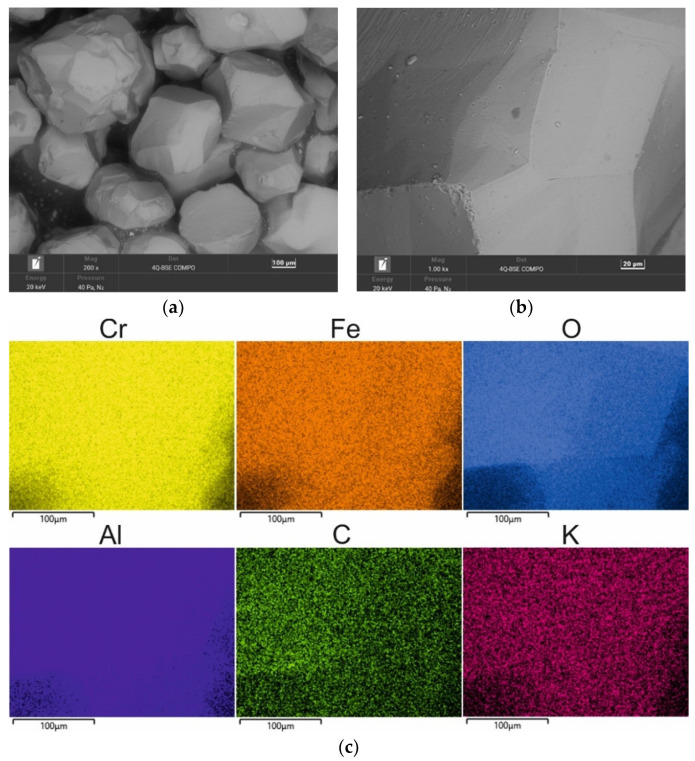
SEM image of chromite sand CS: (**a**) mag. 200×; (**b**) mag. 1000×; (**c**) EDS map of element distribution in the analysed area of chromite sand CS.

A chromite grain matrix (CS) was used in the study—the shape and initial chemical composition of which are shown in Figure 3 (in the form of a SEM image and an EDS map of the element). As can be seen, the chromite sand grains have irregular shapes and many visible angular edges. Table 2 summarises the chemical composition of the chromite sand that was used in the study based on 20 measurements that were made on the grain surface.

**Figure 4:**
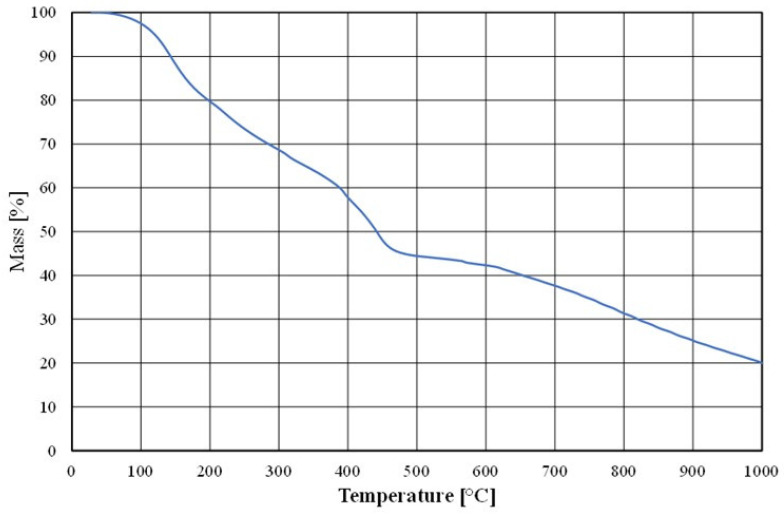
Thermogravimetric analysis of tested material (binder).

To prepare the moulding sand, the Małapanew Ozimek foundry used the above chromite matrix and an alkali-phenolic binder with the trade name of Permabind 440 as well as a Pernamind P8 hardener. A prepared sample of the bonded binder was subjected to thermal (thermogravimetric) analysis; these results are shown in Figure 4.

As can be observed, the binder did not undergo full destruction (i.e., full combustion) up to a temperature of 1000 °C due to the addition of an inorganic alkaline component.

**Figure 5:**
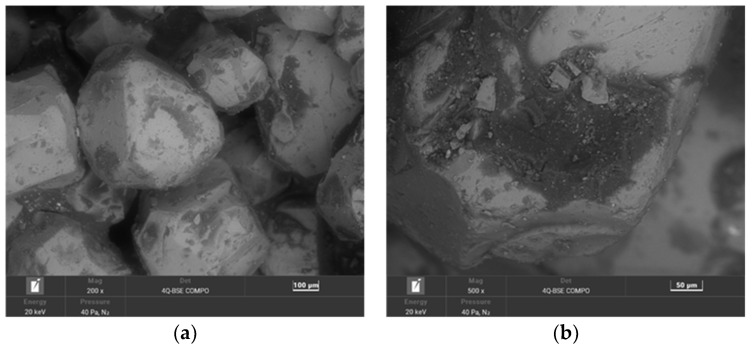

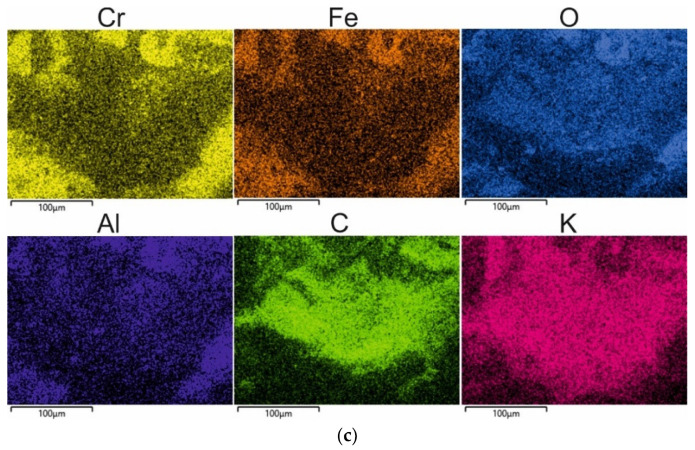
SEM image of spent sand SS: (**a**) mag. 200×; (**b**) mag. 1000×; (**c**) EDS map of element distribution in the analysed area of spent sand SS.

The second material used for the study was moulding sand (SS) from the Małapanew Ozimek foundry. The condition of the tested material is shown in Figure 5 and the chemical composition is shown in Table 3. The blended SEM images show the remaining binder, especially accumulated in the hollows of the matrix, as well as small particles of crushed chromite matrix. The grains are still irregular in shape with sharp edges visible. The maps show the concentration of elements at specific locations on the grain surface. Carbon © and potassium (K) (the primary alkali-phenolic resin-forming elements) were located in the binder residue area on the grain surface (Figure 5b) in the form of bright areas; these indicated the high concentrations of these elements at this location.

Chemical analysis by Scanning Electron Microscopy was performed in the area of the worn alkali-phenolic binder. For each sample, a number of tests were carried out in different micro areas at different magnifications in order to achieve the reproducibility of the results and the suitability of this method for assessing the efficiency of the regeneration processes. The most characteristic high magnifications were selected for publication. The SEM–EDS method was relevant in this case to demonstrate the process of the gradual loss of binder on the surface of the matrix grains (in the form of elemental distribution maps). An SEM–EDS analysis was used to show the trends and assess the impacts of the specific regeneration treatments on the material under study.

A chemical analysis that was performed on the surface of the grain showed the significant presence of both carbon (C) and potassium (K). The corresponding results of the 20 measurements are presented in Table 3 (in the form of a statistical analysis).

**Figure 6:**
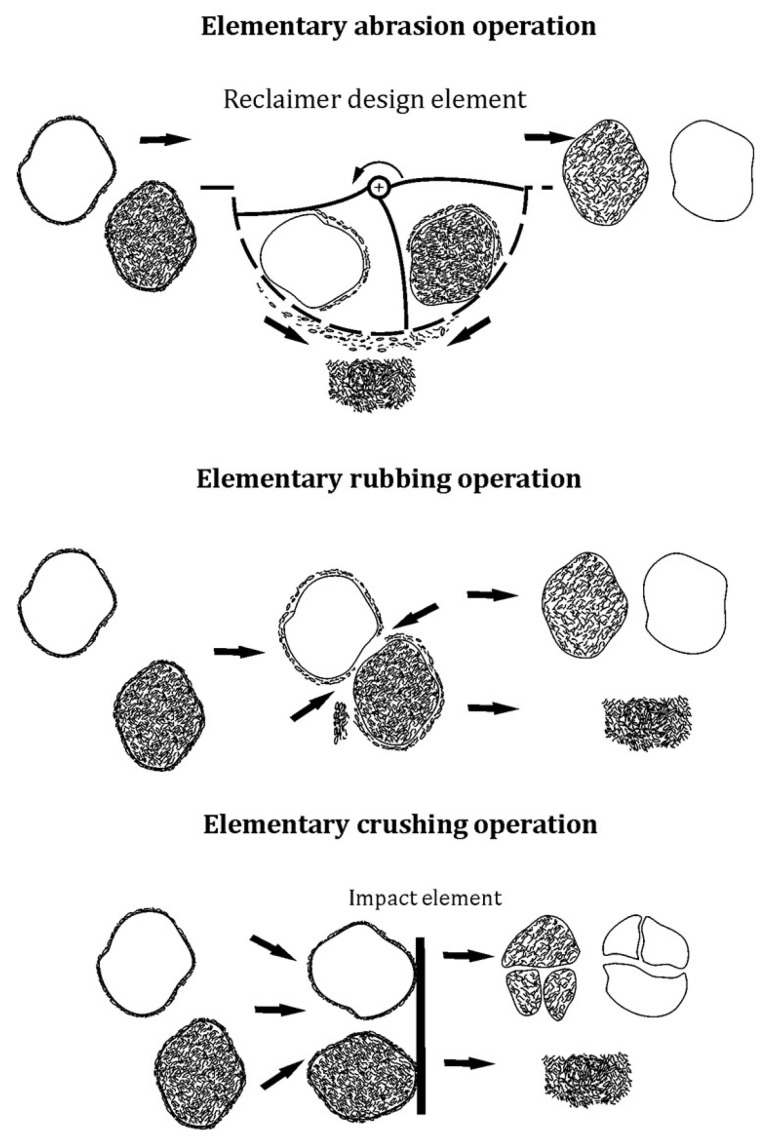
Elementary operations of reclamation process.

After the technological process, the spent sand was subjected to mechanical reclamation in the regenerator after being knocked out of the mould and crushed (shown in Figure 1). During the operation of the device, an extractor was switched on whose role was to extract the dust that was generated during the reclamation. Depending on the machine, mechanical reclamation typically carries out three elementary operations: abrasion, rubbing, and crushing. Figure 6 shows the idea of the operations that are carried out.

Abrasion is a basic procedure of the reclamation process, the purpose of which is to gradually reduce the coating thickness of the bonding material on the surface of the grain as a result of the mechanical action of the machine component. The abrasion of coatings occurs in larger clusters or on individual sand matrix grains in relative motion to the moving or fixed working elements of the machine with which they come into contact [51].

Rubbing is a basic procedure of the reclamation process that consists of a gradual decrease in the thickness of the bonding material coating due to the frictional interaction of the matrix grains against each other. Rubbing occurs in a cluster of loose sand matrix grains that are in relative motion and in direct contact with one another [51].

Crushing is a basic procedure of the regeneration process that involves a sharp reduction in the coating thickness of the bonding material on the grains and a reduction in its particles. Crushing is caused by the pressure of external static or dynamic forces that cause an increase in the contact loads that are transmitted to the coatings by the matrix grains. The forces that are exerted cause the coatings to crack on the grains and the bridges that connect the individual grains. In a loose medium in motion, crushing occurs at the moments of rapid changes in the momentum of the grains that are caused, for example, by their hitting on appropriately shaped device components or impact plates that are horizontally or vertically oriented [51].

The used device mainly carried out two of the three elementary machining operations: abrasion and rubbing. The choice of this reclamation device was based on the limitation of the crushing operation, which could lead to significant fragmentation of the brittle chromite matrix.

As a general criterion for the regenerability of the spent sand matrix, the degree of approximation of the chemical and grain composition, surface morphology, and other properties of the reclaimed sand matrix to the values of the same initial parameters of the sand matrix (obtained with the least possible effort) can be adopted. The quicker a comparable level of undergraded parameters of the reclaimed sand matrix to the initial sand matrix is obtained, the better the regenerability of the mass is consumed by the method [52] as related to the expenditures that are incurred to implement the process.

A number of methods have been proposed for assessing spent sand and reclaimed sand; however, for alkali-phenolic binder sand that is both organic and inorganic, the most appropriate assessment can be identified as ignition loss, sand strength, sieve analysis, and surface morphology with chemical composition analysis in terms of prioritisation.

**Figure 7:**
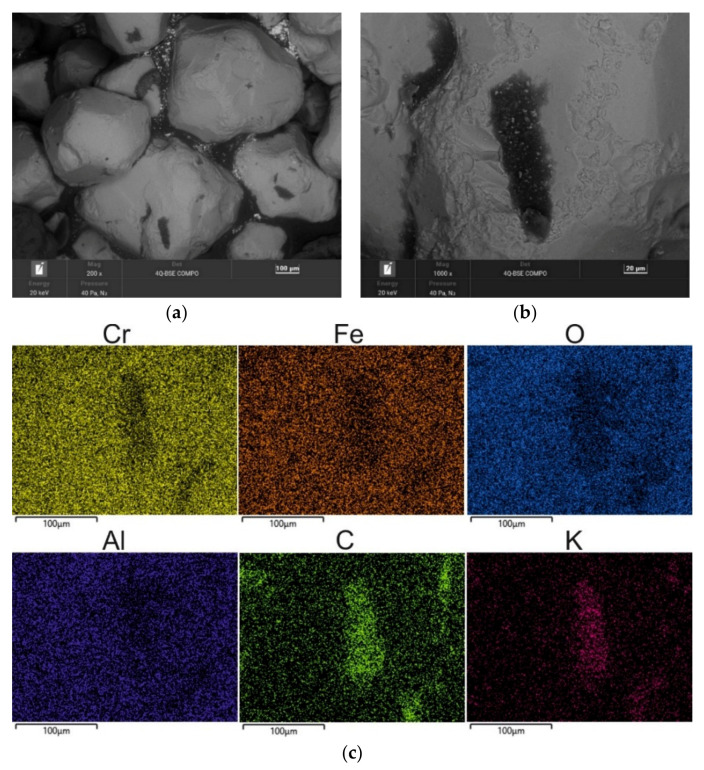
SEM image of 5 min MR5M-reclaimed sand: (**a**) mag. 200×; (**b**) mag. 1000×; (**c**) EDS map of element distribution in the analysed area of MR5M reclaimed sand.

**Figure 8:**
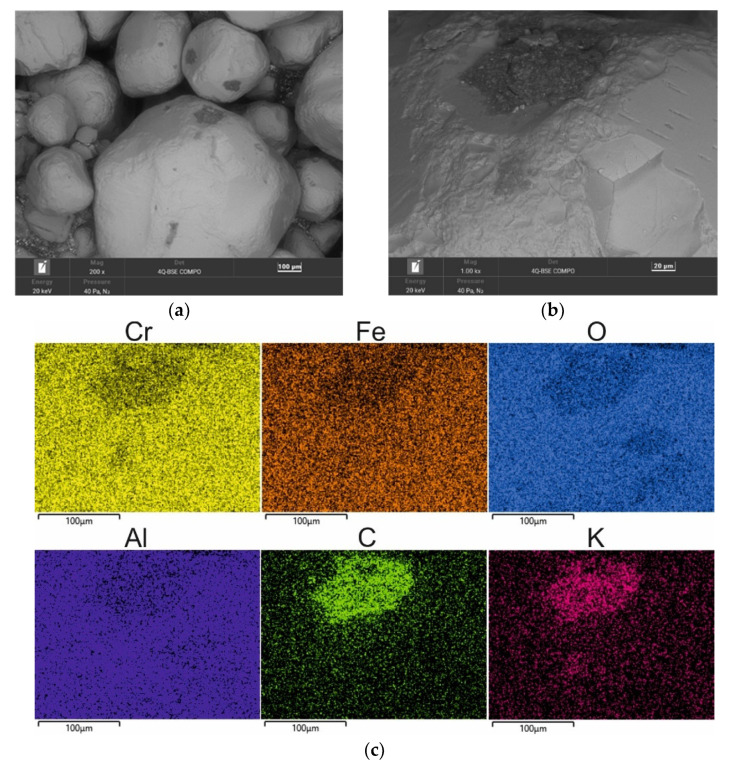
SEM image of 10 min MR10M-reclaimed sand: (**a**) mag. 200×; (**b**) mag. 1000×; (**c**) EDS map of element distribution in the analysed area of MR10M reclaimed sand.

**Figure 9:**
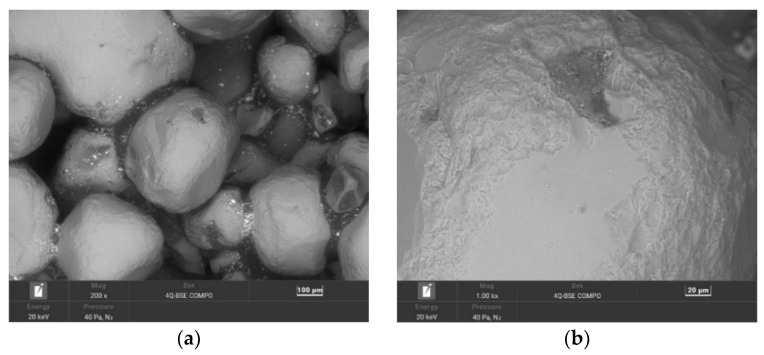

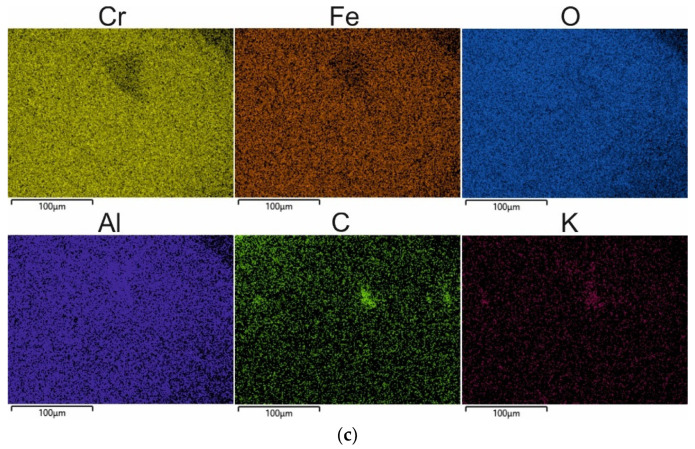
SEM image of 15 min MR15M-reclaimed sand: (**a**) mag. 200×; (**b**) mag. 1000×; (**c**) EDS map of element distribution in the analysed area of MR15M-reclaimed sand.

As a result of the mechanical treatment of the spent sand, grain matrices were obtained for the different reclamation times whose shapes, surface quality, and chemical compositions (in the forms of spectra and maps) are shown in Figure 7, Figure 8 and Figure 9, and the chemical analysis is shown in Table 4, Table 5 and Table 6.

Figure 7 shows the surface morphology of the chromite grain matrix after 5 min of mechanical reclamation. As can be seen, the grains have disturbed surfaces—especially at the edges. At the same time, the spent binder is already only visible in the hollows of the matrix (where the abrasion and rubbing process is hindered). The following maps show the concentration of the binder components (represented by [C] and [K]) precisely in these areas (lighter areas on the map).

The lower binder content on the surface of the matrix after 5 min of reclamation was also confirmed by the chemical analysis (the results of which are presented in Table 4). Significantly lower amounts of carbon and potassium were recorded in the tested material, which indicates the progressive purification of the chromite matrix from the spent binder. During the process, there was an elementary abrasion of the spent binder from the surfaces of the matrix grains against the structural elements of the experimental reclaimer and an elementary rubbing operation that was carried out between the grains that were set in motion. At the same time, the dust was extracted to increase the cleaning effect.

Figure 8 shows chromite matrix grains that were mechanically treated for 10 min. It can be seen that more of the sharp edges were worn away and the grains had increasingly rounded shapes. However, binder residue was still visible in the cavities; this is confirmed by the maps of the analysed constituent elements of the spent binder in the forms of bright fields.

The mechanical reclamation process (which was increased to 10 min as a result of the longer abrasion and rubbing operations) reduced the amounts of the elements that were part of the binder on the grain surface (as shown by the chemical analysis in Table 5).

Extending the mechanical reclamation time by a further 5 min resulted in the further erosion of the grain surfaces (Figure 9) and the removal of even more spent binder residue. The mechanical action was still not effective when the spent binder was in the recesses of the surface (Figure 9). In some studies [53], contaminants were even found to accumulate in the surface irregularities of the grains. The corresponding chemical analysis results for 20 measurements that were taken on the surface of the regenerate after 15 min of mechanical cleaning are shown in Table 6.

**Figure 10:**
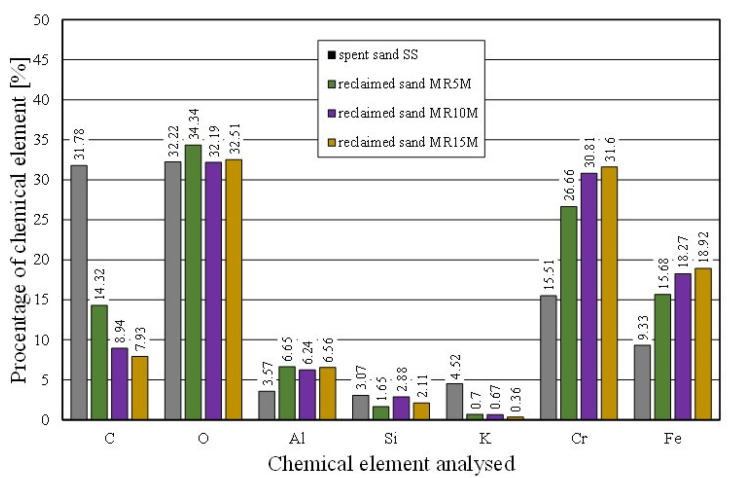
Comparison of chemical composition of tested materials.

**Figure 11:**
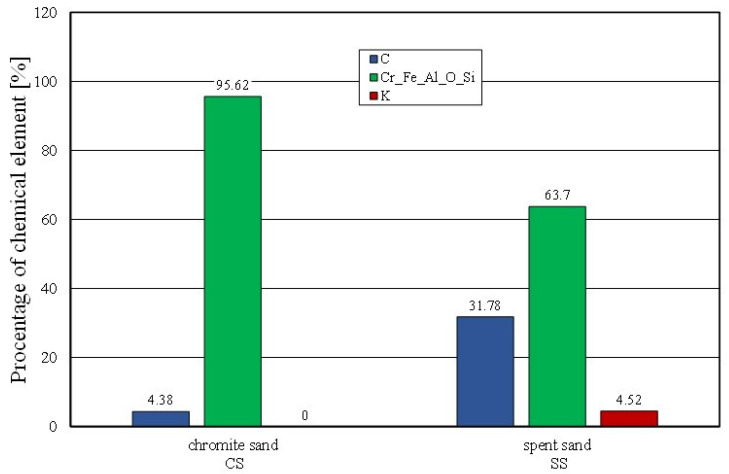
Comparison of chemical composition of fresh chromite sand and spent sand.

**Figure 12:**
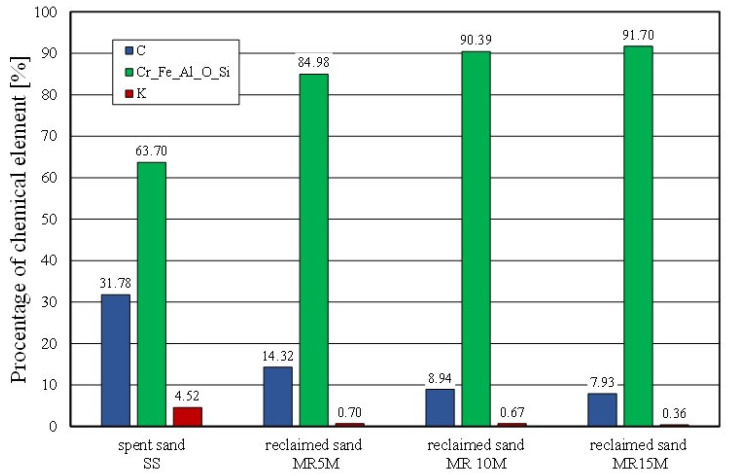
Comparison of chemical composition spent sand and reclaimed sand for different mechanical reclamation times.

The effect of mechanical reclamation is to remove the bound binder from the surface of the matrix grains. Figure 10 summarises the results of the individual chemical elements (their average values), which were determined in selected micro areas on the grain surface, for different intensities of the mechanical treatment. In the case of the Permabind 440 alkali-phenolic binder, the role of reclamation was to remove the organic part (represented by carbon [C]) and the inorganic part (represented by potassium [K]). Therefore, the remaining elements were grouped together for a clearer analysis. The various aspects of the treatment evaluation are shown in Figure 10, Figure 11 and Figure 12.

Figure 11 shows the percentages of the analysed elements (C, K) of the fresh chromite sand (CS) and spent sand (SS) in the sample micro areas. There was a significant amount of carbon (C) and potassium (K) present in the spent sand.

The chemical analysis of the micro areas that are shown in Figure 12 showed a decreasing presence of bond-forming elements on the surface of the matrix grains. The longer the mechanical reclamation time was, the lower the contents of the carbon (C) and potassium (K). The application of 5 min of mechanical treatment and pneumatic grading resulted in a step change in the analysed chemical elements on the surface of the grains.

The morphology of the grain surface, combined with the chemical composition analysis that was carried out, was supplemented by other evaluation criteria in those areas where the residual binder was present. The basic parameter for evaluating the grain matrix was sieve analysis; this made it possible to compare the different grain materials in terms of their granulation. When mechanical reclamation was used, it also made it possible to evaluate the effects of the abrasive treatment that was carried out on the surface of the used moulding sand. This test was a check to ensure that the mechanical action was not too intensive and did not lead to the destruction of the matrix (with a consequent reduction in the grain matrix yield). Sieve analyses were carried out on the starting materials that were accepted for the study (fresh chromite sand [CS] and spent sand [SS]) as well as on the grain matrix after the specified mechanical reclamation times. The results of selected sieve analysis parameters are summarised in Table 7.

During the reclamation of spent sand, the granulometric composition of the matrix should be controlled—especially when it is used repeatedly. Above all, attention should be paid to ensure that, as a result of the treatment processes, there are not too many fine grain fractions (smaller than 0.1 mm) in the obtained reclaimed mass; this is considered to be technologically unsuitable. Their possible presence increases the total specific surface area of the matrix, necessitating the dosage of a larger amount of binder to ensure a certain strength of the moulding sand that is made on the reclaimed mass matrix [52]. Increased binder contents in moulding sand will always generate higher gas emissions (gas-forming), which is not desirable for technological nor environmental reasons. The presence of too fine a fraction or of dust that is generated by the processing that is carried out compromises the permeability of the moulding sand.

The sieve analysis confirmed the mechanical removal of the spent binder from the surfaces of the grains as well as the abrasive action that led to the rounding of the grains (see SEM photo), thus causing reductions in the matrix grains.

The process of recovering the grain matrix from spent sand with synthetic resins (organic, hybrid) involves burning the organic material to form the binder composition. In view of the above, the most important criterion for the effectiveness of the reclamation procedure that is carried out is the determination of the ignition loss of the reclaimed sand.

**Figure 13:**
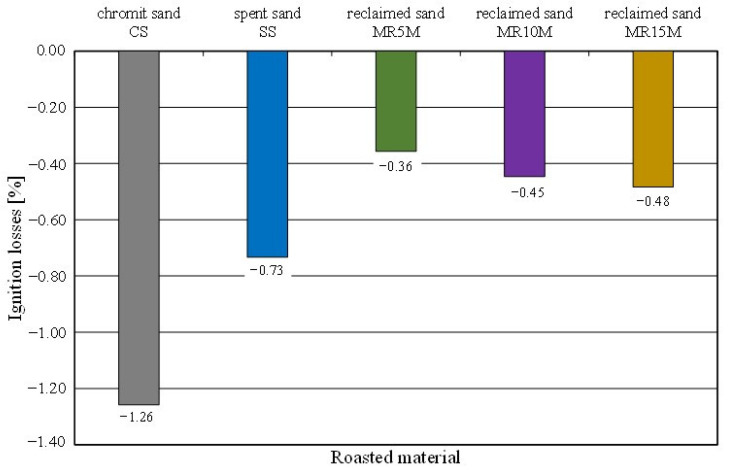
Ignition losses of tested materials.

Tests were carried out on the spent sand (SS) and the regenerates from the different processing times. The obtained results are shown in Figure 13.

When determining this parameter for the spent sand on a quartz matrix with an organic binder [54], positive values were obtained because the mass after the ignition losses was lower due to the burning of the organic resin. In the case of the chromite sand, an increase in the mass of the roasted sample was observed; hence, the ignition losses came out to be negative (Figure 13), despite the firing of the organic part of the binder. All of the tested samples increased in mass after roasting in an oven at 1000 °C for 2 h in the presence of air; this may suggest oxidation of the chromite matrix. The recorded mass change shows a certain regularity. The greatest increase was recorded for the pure chromite matrix. A smaller increase in the mass of the roasted sample was found for the material in which there was the most spent organic binder. In this case, the increase in the weight of the roasted sample with the chromite matrix was compensated by the burned binder. In the cases of the regenerates, mechanical processing abraded the spent binders. The use of extraction during regeneration caused the removal of abrasion products and dust that were created by burning the binder during the pouring of the mould with liquid metal. For increasing reclamation times, a regularity can also be seen. The lower the level of organic material was on the surface of the grain (the result of mechanical processing), the lower the sample mass compensation was for the chromite matrix mass that increased as a result of oxidation.

**Figure 14:**
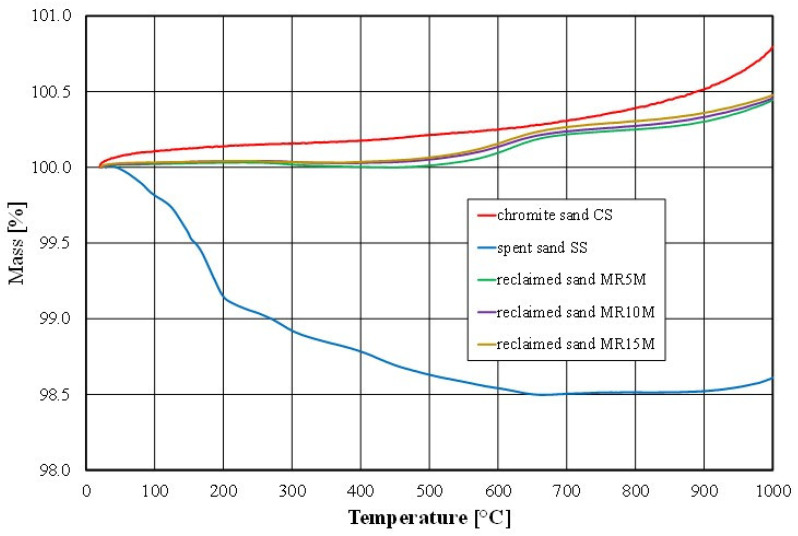
Thermogravimetric analysis of tested materials.

A verification of the observed effect (the cause of the increase in the mass of the sample as a result of the contact of the chromite matrix with the high temperature,) was carried out based on a thermal (thermogravimetric) analysis of fresh chromite sand, spent sand, and the obtained regenerates. The results of the completed analyses are shown in Figure 14.

The thermal analysis indicated that the mass of the fresh chromite matrix increased by about 0.8% under the influence of the temperature. The reason for this phenomenon was the formation of iron oxide (a higher-density material) on the surface of the chromite matrix as a result of its contact with liquid metal (technological process) or as a result of its annealing in a laboratory furnace at above about 700 °C [55] under oxidising conditions (laboratory tests). At the same time, there was a 10% increase in the volume from 600 °C annealing because of the decomposition of the chromite (FeO-Cr_2_O_3_) (density 4500–4800 kg/m^3^) into the mixture of FeO (density 5740 kg/m^3^) and Cr_2_O_3_ (density 5220 kg/m^3^) oxides.

**Figure 15:**
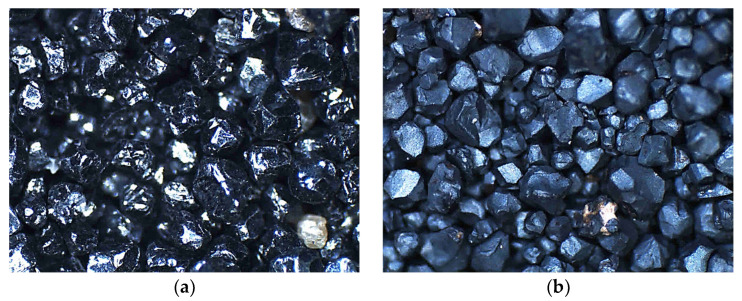
Microscopic images of surface of grain matrix: (**a**) initial chromite sand (mag. 50×); (**b**) chromite sand roasted at 1000 °C (mag. 50×).

This phenomenon resulted in grain cracking and swelling [56]. In view of the above, the change in the volume combined with the change in the density of the newly formed compounds caused an increase in the mass of the chromite matrix that was subjected to the high temperature. Figure 15 shows a photo that was taken with an optical microscope of a fresh chromite matrix (CS) and after roasting at 1000 °C for two hours. The surfaces of the chromite matrix grains after the roasting process were duller, which may indicate the effect of the thermal process in an oxidising atmosphere on the resulting changes in the mass of the test material.

There was a noticeable difference in the results that were obtained between the ignition loss and the thermal analysis for the spent sand (SS); this was due to the different measurement technique. In the case of the ignition loss, a 30 g sample was analysed. For the thermal analysis, we collected 2 g of material where a greater proportion of the sample mass was dust in the case of a highly polydisperse material such as spent sand. However, after the combustion of the organic material (above 600 °C), an increase in the mass of the analysed material sample was also apparent. The thermogravimetric analysis showed the effect of compensating for the increase in sample mass with the combustion of the residual organic material was also apparent in the cases of the regenerates (MR5M, MR10M, and MR15M), as can be observed in the obtained results of the ignition loss.

**Figure 16:**
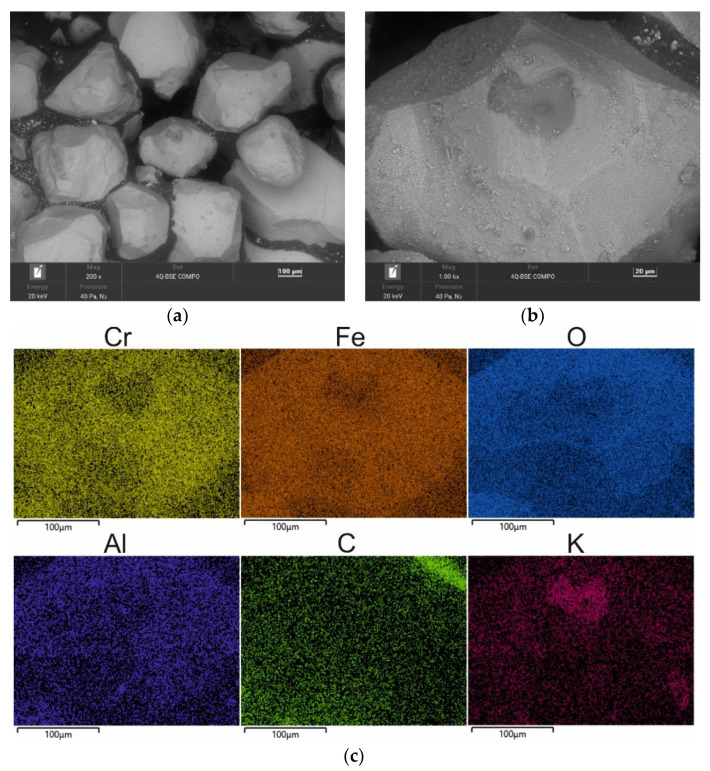
SEM image of spent sand SS after roasting for 2 h at 1000 °C: (**a**) mag. 200×; (**b**) mag. 1000×; (**c**) EDS map of element distribution in the analysed area of spent sand SS after roasting.

As part of the tests, an additional procedure of roasting for 2 h was performed on the material: spent sand samples (SS), and mass samples after the longest mechanical reclamation time (MR15M). The results of the tests that were obtained are shown in Figure 16 and Table 8.

These treatments were the simplest form of thermal reclamation. The thermal reclamation process of spent sand with organic or hybrid binders should be analysed through the prism of polymer breakdown, which occurs during processing and use at elevated temperatures, causing changes in the chemical structure of the polymer as a result of chain scission reactions and oxidation processes, cross-linking of the structure, and changes in the shape and colour of the sample, among others [57]. The aforementioned features were noticeable; therefore, to assess the quality of the obtained reclaimed as a result of the thermal reclamation, a study of the surface morphology of the grain matrix by optical or scanning electron microscopy was used [58,59]. 

When the spent sand (SS) was exposed to 1000 °C, the organic part of the binder was mostly burned off (as can be seen from the carbon content in the chemical analysis that is shown in Table 7). The grains retained their initial angular shapes; at a high magnification, however, it was apparent that there were particles of undusted matrix on the grain surfaces, and changes in the surface quality were visible because of the thermal processes that took place. Figure 16c, which shows the distribution of the elements on the map indicates that potassium K (a component of the inorganic part of the binder) was concentrated in the matrix cavity. This test showed that the application of thermal reclamation alone was not fully effective for the alkali-phenolic binder regarding both the removal of the inorganic part of the binder and the quality of the resulting grain surface.

**Figure 17:**
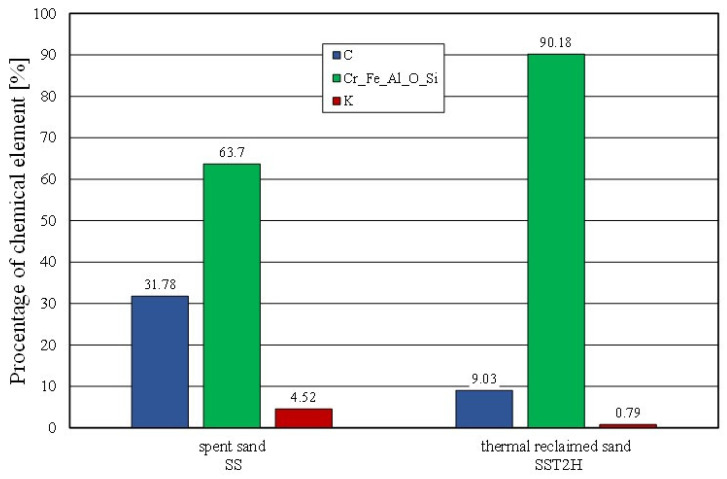
Comparison of chemical composition of spent sand and grain matrix after thermal reclamation.

A further comparison of the elemental contents of the surface of the matrix was carried out for the spent sand and the same material after roasting for two hours at 1000 °C (Figure 17). In this case, the effect of the decrease in the elemental content of the binder was also found. Despite the two hours of roasting and the applied temperature of 1000 °C, a residual carbon content of approximately 9% and a potassium content of approximately 0.8% were determined on the grain surface. As demonstrated by the thermal analysis that was presented earlier, the bonded alkali-phenolic binder was not fully destroyed up to a temperature of 1000 °C.

**Figure 18:**
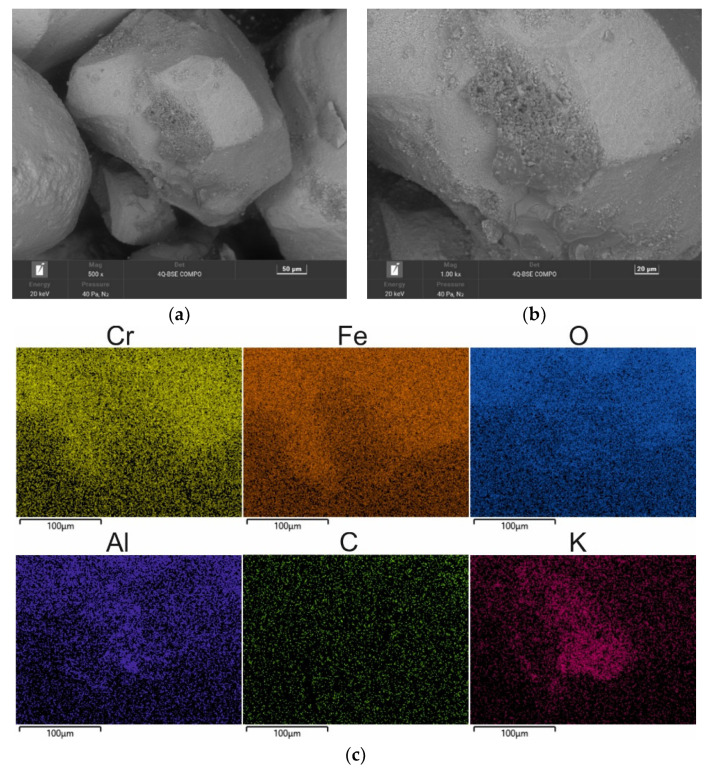
SEM image of 15 min MR15M-reclaimed sand after roasting for 2 h at 1000 °C: (**a**) mag. 200×; (**b**) mag. 1000×; (**c**) EDS map of element distribution in the analysed area of MR15M-reclaimed sand after roasting.

The mechanical regenerate obtained after 15 min, like the spent sand, was subjected to roasting at 1000 °C. Complementing the mechanical reclamation with a thermal treatment further removed the organic part of the binder from the grain surface. The corresponding results are shown in Figure 18 and Table 9. The non-combustible binder residue was still accumulated in the surface irregularities, as illustrated by the map in Figure 18c. On the other hand, in Figure 18, a change in the surface quality of the regenerate so obtained was observed after the introduction of additional heat treatment. The originally smooth grain surface became deformed (cf. Figure 9 and Figure 18), the surface became more wrinkled, and its topography changed. The chromite matrix test confirms the previously signalled phenomenon of an unsatisfactory surface condition after thermal treatment of the spent sand with an alkali-phenolic binder.

**Figure 19:**
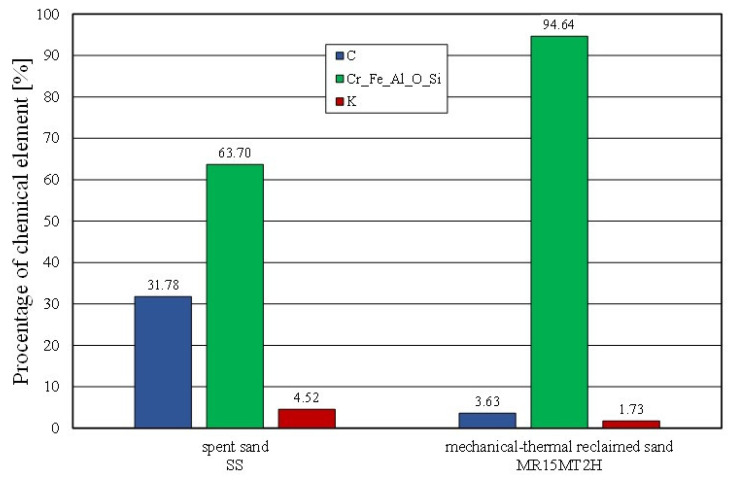
Comparison of chemical compositions of spent sand and grain matrix after mechanical–thermal reclamation.

**Figure 20:**
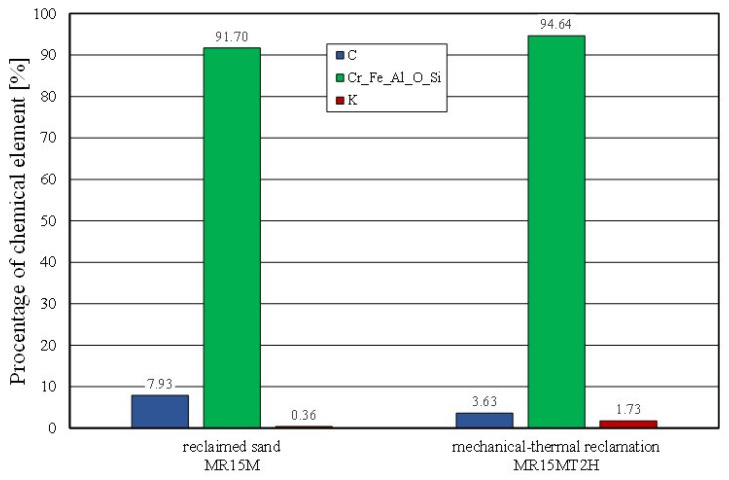
Comparison of chemical compositions of grain matrix after 15 min mechanical reclamation and grain matrix after mechanical–thermal reclamation.

Combined reclamation (e.g., mechanical–thermal reclamation) was applied to mixed moulding sand in which there were both an inorganic binder material (moulding sand) and an organic binder (core mass) [54]. The alkali-phenolic binder mass was partly a hybrid solution, having both organic and inorganic compounds in its composition. Therefore, the interaction of the mechanical and subsequent thermal treatment removed the spent binder to the greatest extent (as illustrated in Figure 19). Thermal reclamation (represented in the study by the roasting of the material samples) resulted in the combustion of the carbon (C), while the residue of potassium (K) (which, as an inorganic element, remained on the grain surface) became visible. This was also confirmed by a comparative analysis of the mechanical reclamation of MR15M and the same material after roasting (MR15M2H). The described changes are shown in Figure 20.

**Figure 21:**
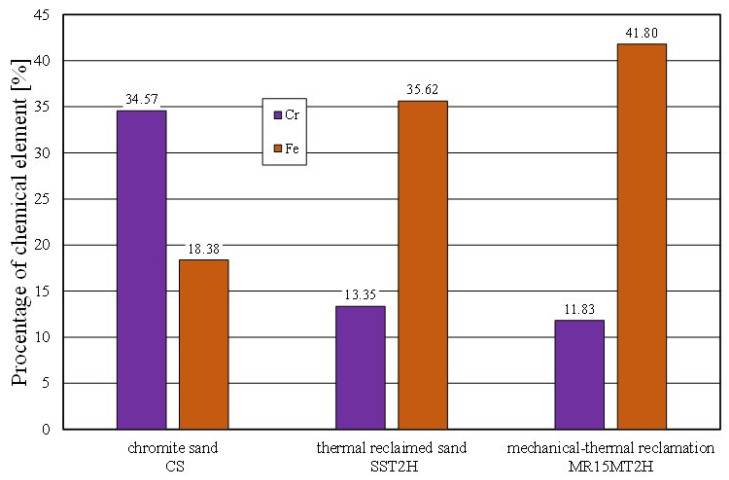
Comparison of chemical composition of grain matrix after thermal reclamation and grain matrix after mechanical–thermal reclamation.

The spent sand with the alkali-phenolic binder (which was not pneumatically graded) was characterised by the presence of impurities on the grain surfaces (not only in the cavities). Obtaining a similar image for the mechanical regenerate that was roasted for 2 h at 1000 °C (MR15M2H) was puzzling. The resulting changes on the surface of the test material were probably the result of transformations that occurred on the surface of the chromite grains above 600 °C. This was confirmed by chemical analysis studies, where an increased iron content was noted on the surface of the grain matrix (SS2H and MR15M2H—materials with the lowest amounts of binder components) as compared to the fresh chromite matrix (CS). The corresponding comparative analysis is shown in Figure 21.

The analyses that are presented here were carried out in order to determine the required intensity of the mechanical action (in this case, the reclamation time) in the RD-6 test reclaimer, which ensured the effective cleaning of the grain matrix from the alkali-phenolic binder. As the tests showed, a time of 15 min was sufficient, as the ignition losses changed little as compared to the reclamation time of 10 min. At the same time, a further prolongation of the mechanical cleaning of the chromite matrix surface resulted in the abrasion of its angular edges (cf. Figure 4b and Figure 7a,b) and, as a consequence, caused a reduction in the grain matrix yield and a reduction in the matrix grain (its fragmentation) (Table 7).

**Figure 22:**
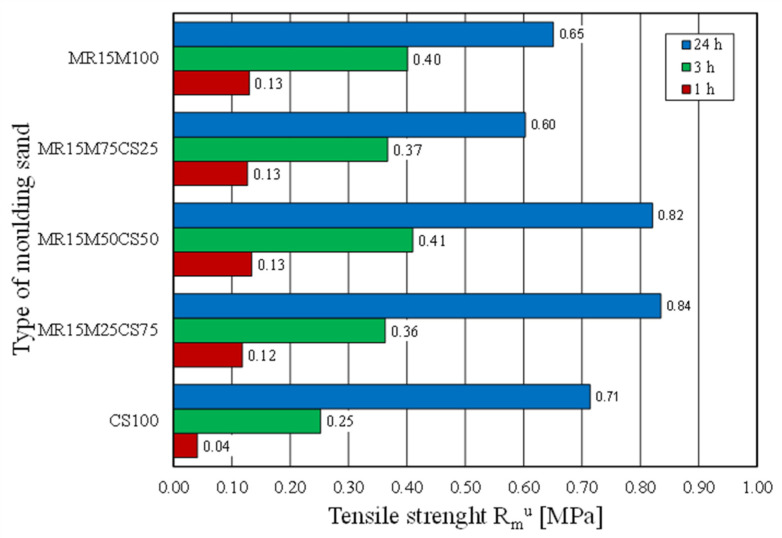
Tensile strength R_m_^u^ of tested moulding sands.

**Figure 23:**
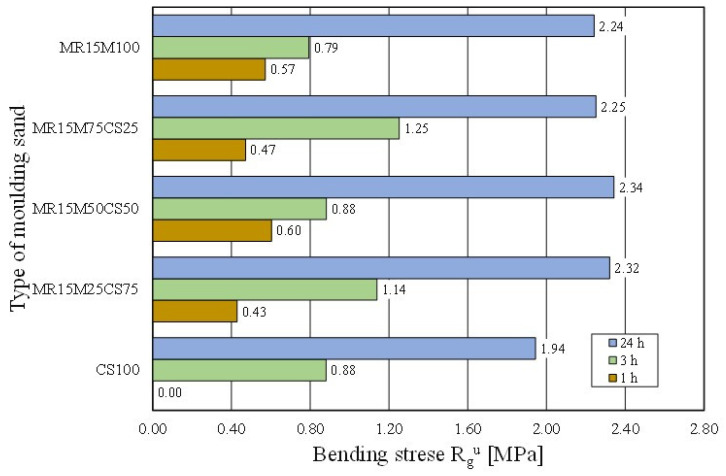
Bending stress R_g_^u^ of tested moulding sands.

The casting moulds were made without using 100% of the reclaimed chromite matrix [15,35]. Therefore, tests of the moulding sand were prepared on the chromite matrix with different proportions of the regenerate. The intention was to determine the ratio of the fresh matrix to the regenerate at which the properties of the moulding sand would not deteriorate. Figure 22 shows how the tensile strength of the moulding sand changed with increasing proportions of the regenerate. The tests showed that a 50% share of the regenerate in the moulding sand enabled comparable results to be obtained with the fresh matrix sand. Another parameter that was tested was the bending strength. These test results are shown in Figure 23. It was concluded from the obtained results that the addition of a regenerate to a fresh matrix does not deteriorate the flexural strength but, on the contrary, improves the parameter that is analysed—reaching a maximum value for a proportion of 50%.

**Figure 24:**
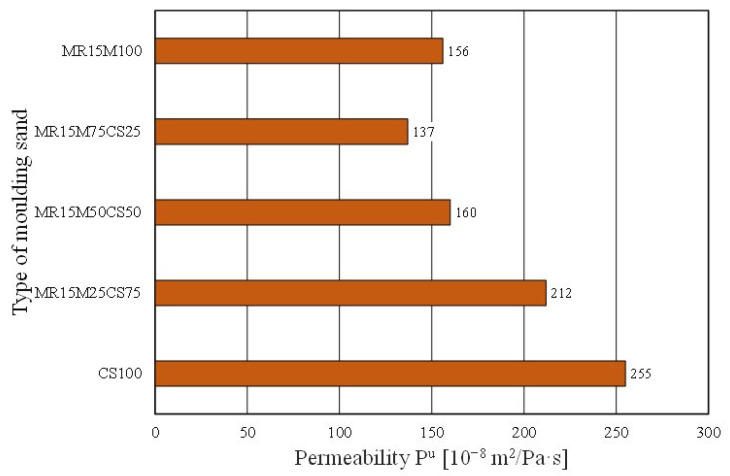
Permeability P^u^ of tested moulding sands.

**Figure 25:**
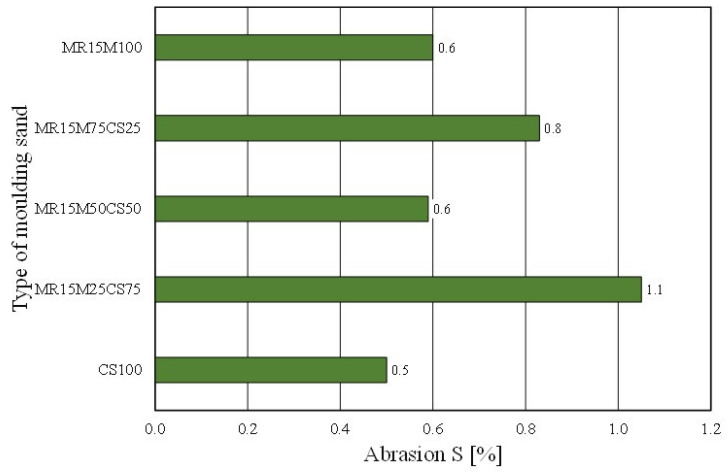
Abrasion S of tested moulding sands.

However, worse results with the share of the regenerate in the grain matrix were recorded for the permeability of the moulding sand (Figure 24). The mixing matrices resulted in the deterioration of the moulding sand’s permeability (even more so as the proportion of the regenerate increased). In the case of the abrasiveness of the tested materials, however, no clear trend was obtained for the effect of the amount of the regenerate in the moulding sand on the studied parameter (Figure 25).

**Figure 26:**
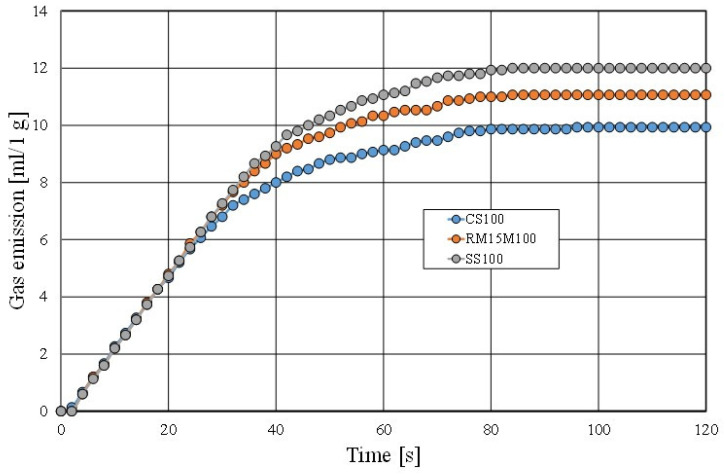
Comparison of gas emissivity of moulding sand.

The final experiment that was performed on the tested materials was to determine the gas emission properties of a moulding sand that was made with a matrix of the fresh chromite sand (CS), the mechanical regenerate (MR15M), and the spent sand (SS). The results of the performed test are shown in Figure 26.

The gas emission capacity of a moulding sand is an important parameter for obtaining good defect-free castings. As can be seen in Figure 26, the more spent binder that remains on the grain matrix, the higher the gas emission of the tested materials. As shown by other studies [52] regarding moulding sands that are prepared on the basis of the multi-cyclic use of the same reclaimed matrix, the saturation of the used binder stabilises for the masses in question at a certain level, which also stabilises the gas emission capacity of the sand and the value of the ignition loss.

## 4. Conclusions

Realising the regeneration process of a spent chromite matrix moulding compound with alkali-phenolic binder requires several important factors to be taken into account; these arise from the machining operations and tests that are carried out:

1. Due to the angular shape of the chromite matrix, elementary regeneration operations (abrasion, rubbing) in the equipment must not occur too intensively, as this leads to the rounding of the chromite matrix grains with a concomitant reduction in their size and, consequently, to an excessive reduction in the grain matrix yield.
2. The reclamation time of spent moulding sand by the mechanical method in a device of a certain intensity is only effective to a limited extent. This is due to the irregular surface of the matrix grains (spent binder remains in the surface irregularities). The analysed parameters of the regenerates (sieve analysis, ignition losses) changed slightly after 10 min and 15 min.
3. For regenerating spent sand on a chromite matrix (which is characterised by brittleness), it is necessary to use non-impact devices in order to not cause the drastic effect of its fragmentation and change the granulometric composition; this may affect the conditions for realising subsequent technological cycles of the casting process (e.g., by worsening the permeability of the sand).
4. The chemical analyses that were performed on the surface of the matrix grains, after the roasting treatment indicate a change in their chemical character, which indicated the thermal destruction of the grain matrix.
5. Due to the thermal instability of the chromite matrix above 600 °C, the selection of an appropriate thermal reclamation temperature is an important issue.
6. The thermal reclamation process mainly leads to the decomposition of the organic polymers, the combustion of the resulting gases, and the burning of the carbon that is produced as a result of the processes that take place. All of these elements of the degradation and destruction of the organic binder mostly take place below 600 °C (which is so critical for the chromite matrix).
7. Given that the alkali-phenolic binder does not completely burn up to a temperature of 1000 °C due to its partially inorganic composition, the use of regeneration temperatures above 600 °C is not economically nor technologically justified (this does not fully remove the binder but does destroy the chromite matrix).
8. Based on the observations that were made, it can be concluded that the sequence of the regeneration procedures is important. Obtaining the clean grain surface of the chromite matrix that is expected for technological reasons indicates that thermal reclamation should be applied first, followed by mechanical reclamation.
9. Studies of the technological parameters of moulding sand that is prepared from a mixture of regenerate and fresh chromite sand have indicated that, in terms of strength, the proportion of regenerate is acceptable in large quantities. When selecting an appropriate proportion of fresh matrix and regenerate, however, one should also take permeability into account, which decreases with increasing amounts of regenerate while the gas emissions that are related to the residue of the spent binder from the previous technological cycle increase.

## Figures and Tables

**Table 1:** Moulding sand mixtures for alkali-phenolic process.

Sand Label	Sand Matrix [wt.%]	Mechanical Reclaim [wt.%]	Resin Permabind [wt.%]	Hardener Permabind P8 [Ratio % of Resin wt.]
CS100	100	0	1.2	25
MR15M25CS75	75	25	1.2	25
MR15M50CS50	50	50	1.2	25
MR15M75CS75	25	75	1.2	25
MR15M100	0	100	1.2	25
SS100	100	0	1.2	25

**Table 2:** Elemental wt.% contents of chromite sand CS in the analysed area (in Figure 3b).

Element wt.%	C	O	Al	Si	Cr	Fe
Max	6.41	37.00	8.42	5.02	37.25	20.94
Min	3.58	28.48	5.94	1.86	29.22	16.9
**Average**	**4.38**	**32.45**	**7.49**	**2.73**	**34.57**	**18.38**
Standard Deviation σ	0.88	1.87	0.67	0.93	1.97	1.08

**Table 3:** Elemental wt.% contents of spent sand SS in the analysed area (in Figure 5b).

Element wt.%	C	O	Al	Si	K	Cr	Fe
Max	38.56	37.00	7.45	9.09	6.25	20.15	11.52
Min	22.32	27.60	2.84	1.61	2.43	12.16	7.25
**Average**	**31.78**	**32.22**	**3.57**	**3.07**	**4.52**	**15.51**	**9.33**
Standard Deviation σ	4.44	2.01	0.94	1.65	1.12	2.20	1.28

**Table 4:** Elemental wt.% contents of 5 min MR5M reclaimed sand in the analysed area (in Figure 7b).

Element wt.%	C	O	Al	Si	K	Cr	Fe
Max	16.67	35.36	7.13	1.74	0.85	28.87	16.37
Min	12.36	33.00	6.17	1.58	0.50	25.08	14.75
**Average**	**14.32**	**34.34**	**6.65**	**1.65**	**0.70**	**26.66**	**15.68**
Standard Deviation σ	1.49	0.74	0.28	0.07	0.14	1.20	0.48

**Table 5:** Elemental wt.% contents of 10 min MR10M-reclaimed sand in the analysed area (in Figure 8b).

Element wt.%	C	O	Al	Si	K	Cr	Fe
Max	14.51	34.47	7.17	4.34	0.94	34.05	19.47
Min	5.44	29.82	5.59	1.88	0.26	26.94	16.43
**Average**	**8.94**	**32.19**	**6.24**	**2.88**	**0.67**	**30.81**	**18.27**
Standard Deviation σ	2.58	1.74	0.38	0.95	0.20	2.37	0.88

**Table 6:** Elemental wt.% contents of 15 min MR15M-reclaimed sand in the analysed area (in Figure 9b).

Element wt.%	C	O	Al	Si	K	Cr	Fe
Max	13.86	39.89	7.94	2.98	0.59	37.19	22.18
Min	2.21	25.36	5.60	1.48	0.06	24.09	14.43
**Average**	**7.93**	**32.51**	**6.56**	**2.11**	**0.36**	**31.60**	**18.92**
Standard Deviation σ	3.17	5.59	0.74	0.44	0.15	4.57	2.65

**Table 7:** Parameters of tested grain matrices as determined by sieve analysis.

Material Label	Mean Grain Size d_a_ mm	Sieve Analysis Parameter	Uniformity Index J %	Surface Area S_t_ m^2^/kg
CS	0.36	0.20/0.32/0.40	64	4.24
SS	0.40	0.20/0.40/0.32	57	4.33
MR5M	0.33	0.20/0.32/0.40	61	5.04
MR10M	0.32	0.20/0.32/0.40	58	5.57
MR15M	0.32	0.20/0.40/0.32	56	5.72

**Table 8:** Elemental wt.% contents of spent sand SS after roasting for 2 h at 1000 °C in the analysed area (in Figure 16b).

Element wt.%	C	O	Al	Si	K	Cr	Fe
Max	11.78	33.96	3.55	6.34	1.49	14.05	39.79
Min	6.59	29.84	2.80	2.94	0.53	12.96	33.15
**Average**	**9.03**	**32.87**	**3.13**	**5.21**	**0.79**	**13.35**	**35.62**
Standard Deviation σ	1.20	1.19	0.19	1.13	0.24	0.30	1.70

**Table 9:** Elemental wt.% contents of 15 min MR15M-reclaimed sand after roasting for 2 h at 1000 °C in the analysed area (in Figure 18b).

Element wt.%	C	O	Al	Si	K	Cr	Fe
Max	7.59	36.79	4.39	6.16	2.61	13.25	46.69
Min	1.88	29.2	2.97	2.74	0.68	10.97	36.77
**Average**	**3.63**	**32.93**	**3.66**	**4.42**	**1.73**	**11.83**	**41.80**
Standard Deviation σ	1.53	2.84	0.38	1.08	0.57	0.81	3.43

## Data Availability

The data that support the findings of this study are available from the corresponding authors (M.Ł., A.G.-K., D.D., K.K.) upon reasonable request.
